# Population Pharmacokinetics of Palbociclib and Its Correlation with Clinical Efficacy and Safety in Patients with Advanced Breast Cancer

**DOI:** 10.3390/pharmaceutics14071317

**Published:** 2022-06-21

**Authors:** Perrine Courlet, Evelina Cardoso, Carole Bandiera, Athina Stravodimou, Jean-Philippe Zurcher, Haithem Chtioui, Isabella Locatelli, Laurent Arthur Decosterd, Léa Darnaud, Benoit Blanchet, Jérôme Alexandre, Anna Dorothea Wagner, Khalil Zaman, Marie Paule Schneider, Monia Guidi, Chantal Csajka

**Affiliations:** 1 Centre for Research and Innovation in Clinical Pharmaceutical Sciences, Lausanne University Hospital and University of Lausanne, 1011 Lausanne, Switzerland; perrine.courlet@chuv.ch (P.C.); monia.guidi@chuv.ch (M.G.); 2 Precision Oncology Centre, Department of Oncology, Lausanne University Hospital and University of Lausanne, 1011 Lausanne, Switzerland; 3 School of Pharmaceutical Sciences, University of Geneva, 1206 Geneva, Switzerland; evelina.cardoso@unige.ch (E.C.); carole.bandiera@unisante.ch (C.B.); marie.schneider@unige.ch (M.P.S.); 4 Institute of Pharmaceutical Sciences of Western Switzerland, University of Geneva, 1206 Geneva, Switzerland; 5 Center for Primary Care and Public Health (Unisanté), University of Lausanne, 1011 Lausanne, Switzerland; isabella.locatelli@unisante.ch; 6 Breast Center, Department of Oncology, Lausanne University Hospital and University of Lausanne, 1011 Lausanne, Switzerland; athina.stravodimou@chuv.ch (A.S.); jean-philippe.zurcher@chuv.ch (J.-P.Z.); dorothea.wagner@chuv.ch (A.D.W.); khalil.zaman@chuv.ch (K.Z.); 7 Service of Clinical Pharmacology, Lausanne University Hospital and University of Lausanne, 1011 Lausanne, Switzerland; haithem.chtioui@chuv.ch (H.C.); laurentarthur.decosterd@chuv.ch (L.A.D.); 8 Department of Pharmacokinetics and Pharmacochemistry, Cochin Hospital, AP-HP, CARPEM, AP-HP, CARPEM, 75014 Paris, France; darnaud.lea@orange.fr (L.D.); benoit.blanchet@aphp.fr (B.B.); 9 UMR8038 CNRS, U1268 INSERM, Faculty of Pharmacy, University of Paris, PRES Sorbonne Paris Cité, CARPEM, 75006 Paris, France; 10 Department of Medical Oncology, Cochin University Hospital, Assistance Publique-Hôpitaux de Paris, 75014 Paris, France; jerome.alexandre@aphp.fr

**Keywords:** palbociclib, pharmacokinetics, neutropenia, progression-free survival

## Abstract

Neutropenia is the most frequent dose-limiting toxicity reported in patients with metastatic breast cancer receiving palbociclib. The objective of this study was to investigate the pharmacokinetic–pharmacodynamic (PK/PD) relationships for toxicity (i.e., absolute neutrophil count, ANC) and efficacy (i.e., progression-free survival, PFS). A semi-mechanistic PK/PD model was used to predict neutrophils’ time course using a population approach (NONMEM). Influence of demographic and clinical characteristics was evaluated. Cox proportional hazards models were developed to evaluate the influence of palbociclib PK on PFS. A two-compartment model with first-order absorption and a lag time adequately described the 255 palbociclib concentrations provided by 44 patients. The effect of the co-administration of proton-pump inhibitors in fasting conditions increased palbociclib clearance by 56%. None of the tested covariates affected the PD parameters. Model-based simulations confirmed the concentration-dependent and non-cumulative properties of palbociclib-induced neutropenia, reversible after treatment withdrawal. The ANC nadir occurred approximately at day 24 of each cycle. Cox analyses revealed a trend for better PFS with increasing palbociclib exposure in older patients. By characterizing palbociclib-induced neutropenia, this model offers support to clinicians to rationally optimize treatment management through patient-individualized strategies.

## 1. Introduction

Breast cancer is the most frequent cancer in women, with 2.3 million diagnoses and 685′000 deaths globally in 2020 [1]. In the last decade, the progresses in molecular biology allowed the development of very specific targeted therapies for the different breast cancers subtypes [2,3]. Palbociclib is an oral, highly selective inhibitor of cyclin-dependent kinases (CDK) 4 and 6 involved in cellular proliferation. It is approved for the treatment of hormone receptor (HR)–positive, human epidermal growth factor receptor 2 (HER2)–negative locally advanced or metastatic breast cancer in combination with fulvestrant, tamoxifen, or an aromatase inhibitor [4]. The efficacy and safety of palbociclib were demonstrated in randomized, double-blind, placebo-controlled Phase III trials in combination with fulvestrant [5] or letrozole [6].

Palbociclib is administered at a standard dose of 125 mg once daily for 21 consecutive days followed by 7 days OFF-treatment (complete cycle of 28 days). Its pharmacokinetics (PK) is characterized by a large inter-individual variability [7]. High gastric pH influences its absorption due to a decrease in palbociclib solubility [8]. In addition, its metabolism depends on the activity of cytochrome P450 (CYP) 3A and sulfotransferase 2A1, making palbociclib a potential victim of drug–drug interactions. Other factors, such as demographic characteristics (e.g., body weight, race), comorbidities (e.g., hepatic impairment), and medication adherence, may influence palbociclib PK. As a consequence of high PK variability, subtherapeutic drug exposure may result in treatment failure, as observed for other oral anticancer drugs [9,10]. Data from the manufacturer suggest a trend for better progression-free survival (PFS) with increasing palbociclib exposure [4]. A recent study demonstrated that palbociclib doses did not significantly affect PFS [11]; however, it did not investigate the plasma concentration’s impact on it. Conversely, supratherapeutic concentrations may cause drug adverse events or toxicity. Although generally well tolerated, the most commonly reported toxicity under palbociclib treatment is neutropenia, frequently resulting in dose reduction and/or treatment discontinuation [6,12]. Data from the manufacturer suggest a more severe neutropenia with increasing palbociclib exposure [4]. These results have been recently confirmed in a recent real-world data analysis [13]. However, to our knowledge, no real-world study reported on both PK–toxicity and PK–efficacy results in the same population. 

The aim of this study was to characterize the PK/PD relationships of palbociclib in a real-world setting of breast cancer patients to assess the influence of palbociclib PK on the absolute neutrophil count (ANC) time profile and PFS. The resulting PK/PD model was used to simulate the effect of palbociclib dosing regimen on ANC kinetics. An optimal palbociclib exposure limiting the risk of severe neutropenia was finally proposed.

## 2. Material and Methods

### 2.1. Study Population and Data

Palbociclib samples were collected as part of the OpTAT (Optimizing oral Targeted Anticancer Therapies, ClinicalTrials.gov: NCT04484064) study implemented at the University Hospital of Lausanne (CHUV, Lausanne, Switzerland) and at the Center of Primary Care and Public Health (Unisanté, Lausanne, Switzerland) and approved by the Human Research Ethics Committee of the Canton de Vaud (Lausanne, Switzerland) [14]. All included patients were ≥ 18 years old, diagnosed with an advanced stage breast cancer, and provided written informed consent. Blood samples taken at routinely scheduled visits plus extensive collection over a 10–12 h dosing interval were gathered during the study. Collected samples were centrifuged, and plasma was stored at −80 °C allowing its conservation until drug level measurements.

For all patients, palbociclib dosing history was collected at each blood sampling and was retrieved from patient’s medical records. Moreover, information was reconciled with data collected in the randomized controlled medication adherence study [14,15] in a subset of patients. 

ANC was directly retrieved from the patients’ medical records up to the date of the last PK observation. The most recent ANC value in the three months before palbociclib initiation was retained as the base value. Blood sampling for ANC measurement was performed either concomitantly or separately from that for palbociclib quantification.

To retrieve informative data on renal and hepatic functions, a time lapse of 15 days around the date of PK or ANC measurements was considered. Demographic and clinical characteristics were collected at the closest sampling date. The administration of exogenous granulocyte colony-stimulating factors (G-CSFs), a treatment preventing palbociclib-induced neutropenia, was also considered. ANC data were excluded from the analysis for patients with G-CSF administration from the date of injection to 7 days later, considering the information from the European Medicine Agency stating that “discontinuation of G-CSF therapy usually results […] in a return to normal levels in 1 to 7 days”. 

### 2.2. Analytical Methods

Palbociclib plasma concentrations were measured using high-performance liquid chromatography coupled to tandem mass spectrometry at the Laboratory of Clinical Pharmacology of the Lausanne University Hospital. The chromatographic separation was performed on a column XSelect HSS T3 2.1 × 75 mm, 3.5 µm (Waters^®^, Milford, MA, USA) using a mobile phase composed of a gradient of ammonium acetate 2 mM (+0.1 formic acid) and acetonitrile (+0.1 formic acid). Palbociclib was detected by electrospray triple-stage quadrupole mass spectrometry and quantified using the calibration curves with stable isotope-labeled internal standard palbociclib-D8 (Alsachim, Illkirch-Graffenstaden, France). The assay showed appropriate inter-day and intra-day precision for the quantification of palbociclib (coefficient of variation [CV] 0.6–5.9% and 5.7–8.4%, respectively) and accuracy (−1.2% to 5.4%). The method was validated over the clinically relevant concentration range, i.e., from 0.5 to 500 ng/mL.

ANC was measured either by the Laboratory of Clinical Chemistry or by point-of-care tests (POCTs) in the service of Medical Oncology. Both results were integrated in the dataset, and POCT measures were flagged.

### 2.3. Pharmacokinetic Model and Exposure–Toxicity Relationship

Pharmacokinetic and exposure–toxicity models were developed using non-linear mixed effect modeling (NONMEM, version 7.4.3; ICON Development Solutions, Ellicott City, MD, USA), supplemented with the Perl-speaks-NONMEM toolkit (PsN, version 4.8.0) and Pirana interface (version 2.9.7) [16,17]. Data management and statistical and graphical analyses were performed using R (version 4.0.2, https://www.r-project.org (accessed on 4 January 2021)). 

#### 2.3.1. Pharmacokinetic Model

The palbociclib dosing history during the previous cycle and the current one for each palbociclib concentration were integrated in the PK database using the ADDL function in NONMEM. One- and two-compartment models with first- or zero-order absorption, potentially including an absorption lag time were tested. Inter-individual variability was modeled exponentially and tested sequentially on all parameters. Additive, proportional, and mixed models for residual error were compared.

#### 2.3.2. Exposure–Toxicity Relationship

The proliferation, maturation, and homeostatic regulation of neutrophils were mimicked by a series of 5 compartments, as described in the model developed by Friberg et al. [18]. This structural model was directly applied to our data. The first compartment represented stem cells and proliferating precursor cells in the bone marrow with the systematic renewal of proliferative cells’ production modeled by the rate constant *k_prol_*. This compartment was followed by three transit compartments to account for the delay between proliferation and maturation according to the first-order rate constant *k_tr_*, defined as $\frac{n+1}{MTT}$, where *MTT* is the mean transit time of neutrophils. The last compartment characterized the blood circulation with the neutrophil elimination rate constant *k_circ_*. The rate constants *k_prol_*, *k_tr_*, and *k_circ_* were assumed to be equal, as in the original model. In order to mimic physiology by increasing the proliferation rate of progenitor cells in the bone marrow when circulating neutrophils are low, a feedback mechanism was modeled as ${\left(\frac{Base}{Circ_{t}}\right)}^{\gamma },$ where *Circ_t_* represents the circulating cells at a given time, and *Base* is the baseline circulating cell count before any palbociclib administration.

Based on previously published models [13,19], palbociclib’s effect was implemented on the proliferation compartment. A linear (Equation (1)) and an *E_max_* (Equation (2)) functions were tested and compared to describe the palbociclib-induced neutropenic effect:

(1) $$E_{palbo}=Slope\times C_{palbo}$$

(2) $$E_{palbo}=\frac{E_{max}\times C_{palbo}}{EC_{50}+C_{palbo}}$$

with *C_palbo_* being the model-predicted palbociclib concentration in the central compartment, *Slope* being the sensitivity to drug myelotoxicity, *E_max_* being the maximum effect of palbociclib on *k_prol_*, and *EC*_50_ being the palbociclib concentration that produced a 50% effect.

ANCs were log-transformed before the analysis, and an additive error on the log scale was used to describe residual variability. Owing to long computation runtime, neutropenia model development was first performed by fixing the PK parameters that were estimated together with PD parameters in the final model.

#### 2.3.3. Covariate Analysis

Potential associations between post hoc individual parameter estimates and demographic or clinical characteristics were first visually explored and then tested in the model. The covariates investigated for their impact on palbociclib PK were as follows: age, body weight, body mass index, aspartate aminotransferase (AST), alanine aminotransferase (ALT), total bilirubin (BILT), albumin, alkaline phosphatase (ALK), administration under fasting conditions, co-administration of CYP3A4 inhibitors or inducers, co-administration of proton-pump inhibitor (PPI), and cycle number on apparent clearance (CL/F); fasting conditions and PPI co-administration on absorption rate constant (k_a_); fasting conditions on lag time (ALAG); and body weight on apparent central volume of distribution (V_c_/F). Although renal elimination is not the major route of palbociclib elimination, creatinine (CRT) and estimated glomerular filtration rate (eGFR, estimated with the Cockroft and Gault formula) were tested on palbociclib CL/F since a recent study reported an association with palbociclib exposure [13]. The characteristics available for testing their effect on PD parameters were as follows: age, body weight, ALK, BILT, gamma-glutamyltransferase (GGT), albumin, AST, ALT, eGFR, association with fulvestrant, chemotherapy in the previous treatment line, and POCT measurement. Concomitant treatment with fulvestrant was tested since neutropenia was also reported in patients receiving fulvestrant alone (although to a lower extent compared to palbociclib) [20]. Despite high inter-individual variability on EC_50_, covariates were not tested on this parameter and were substituted by covariates on E_max_ as recommended in good practices for exposure–response analyses [21]. Baseline values of continuous covariates were tested on the base ANC parameter while time-varying values were tested on E_max_. Continuous covariates were centered and normalized on their median value and tested by linear or allometric functions as appropriate. Missing data were imputed to the population median value. Categorical covariates were coded as 0 or 1, and missing data were first treated as an additional category and then regrouped according to their parameter estimates.

#### 2.3.4. Parameter Estimation and Model Selection

The first-order conditional estimation method with interaction (FOCEI) was used for model fitting with the ADVAN4 TRANS4 subroutine for the PK model and the ADVAN6 subroutine defining a system of ordinary differential equations for the PK/PD model. Models were selected based on the improvement in standard goodness-of-fit plots, precision of parameter estimates and eta shrinkage. Nested models were compared on the basis of change in the objective function value (∆OFV ≤ −3.84, *p* ≤ 0.05 for one additional parameter considered statistically significant), while Akaike’s criteria (AIC) was employed for non-hierarchical models (drop in AIC of 2 considered relevant [22]). The covariate analysis was performed by forward inclusion (significant level for ∆OFV ≤ −3.84, *p* ≤ 0.05) followed by backward deletion (significant level for ∆OFV ≥ 7.88, *p* ≤ 0.005).

#### 2.3.5. Model Evaluation

An external validation of the PK model was conducted using data from the OpTAT study combined with palbociclib measurements from an independent group of patients followed at the Cochin Hospital in Paris. Plasma concentrations were predicted by post hoc Bayesian forecasting with the MAXEVAL = 0 option in NONMEM. Both bias (mean prediction error, *MPE*, with its 95% confidence interval (CI_95%_)) and precision (root mean square prediction error, *RMSE*) were computed as follows to evaluate the predictive performance of the model:

(3) $$MPE=\exp\left(\frac{\sum^{\text{​}}C_{pred}-C_{obs}}{N}\right)-1$$

(4) $$RMSE=\sqrt{\frac{\sum^{\text{​}}{\left(C_{pred}-C_{obs}\right)}^{2}}{N}}-1$$

with *C_pred_* and *C_obs_* denoting log-transformed individual predictions and observed concentrations, respectively, and *N* representing the number of observations [23].

The final PK/PD model was evaluated with a non-parametric bootstrap using 502 replicates of the initial dataset. The generated median parameters and their CI_95%_ were compared to the final model estimates. In addition, prediction-corrected visual predictive checks (pc-VPCs) were performed to diagnose potential model misspecification by visually comparing observed concentrations with prediction percentiles [24].

#### 2.3.6. Model-Based Simulations

##### PPI Effect

Simulations based on the final PK model in 1000 individuals per group allowed the comparison of palbociclib disposition after administration during a meal without PPI or in fasting conditions with PPI.

##### Neutropenia Characteristics

Hematotoxicity was classified according to their grade in the Common Terminology Criteria for Adverse Events version 5 (i.e., 1 G/L > ANC > 0.5 G/L for grade 3 and ANC < 0.5 G/L for grade 4) [25]. The reversibility of neutropenia was evaluated after dose reduction or treatment interruption. Time to recovery to base ANC value was also computed for a scenario of three palbociclib cycles with a standard administration schedule. The incidence and nadir of neutropenia grade ≥ 3 as well as the time to nadir after treatment initiation were calculated for each simulated scenario. Patients were also divided into four equal subgroups according to their baseline ANC values, and neutropenia characteristics were compared between each group.

##### Influence of Palbociclib Dosage Regimen on Neutropenia

PK/PD final model simulations were performed in 1000 individuals receiving palbociclib during a meal (as recommended) without PPI under several dosage regimens to evaluate their influence on neutropenia. Both changes in palbociclib daily dose (i.e., 100 or 75 mg) and extension of OFF phases (i.e., 14 days OFF) compared to the standard regimen (i.e., 125 mg, 21 days ON/7 days OFF) were simulated. 

##### Exposure Threshold to Limit the Risk of Severe Neutropenia

Finally, to simulate the proportion of patients with at least one grade ≥ 3 or grade 4 neutropenia, 1000 PK profiles of patients receiving 125 mg palbociclib under a standard administration schedule were first simulated to compute the area under the curve from 0 to 24 h (AUC_0–24_) dividing the dose by the individual CL/F. Patients were grouped according to their AUC_0–24_ in steps of 100 ng·h/mL, and the individual PK parameters of an average patient in each group were retrieved within each interval and used for the PK/PD simulations. Then, the simulations allowed 1000 ANC time courses to be obtained during 3 cycles for each retained patient and the proportion of patients with at least one grade ≥ 3 or grade 4 ANC value for each AUC_0–24_ interval was calculated. An empirical value of 10% of patients at risk of severe neutropenia was retained as a maximum threshold not to be exceeded. This value was selected to minimize the risk of neutropenia to a clinically acceptable proportion of patients.

### 2.4. Exposure–Response Relationship

Palbociclib AUC_0–24_ was computed for each day from day 1 of treatment initiation to the progression date (date of the radiologic evaluation at which progression was evidenced) or to the date of treatment discontinuation (for toxicity reasons) or to the date of the last follow up or to the endpoint date (if treatment was pursued after December 2020), including OFF-treatment periods. To consider the time lapse between radiologic exams (i.e., every 3 months), mean cumulative AUC_0-24_ over 90 days (AUC_cum90_) was then predicted. This variable was selected as the exposure metric to be tested as a time-dependent covariate for its influence on PFS. In consideration of the limited number of patients progressing (n = 29), we only included age at treatment initiation as an additional covariate, with an interaction term between age and exposure. Cox proportional hazards models were estimated in R (2021) to evaluate the effect of these covariates on PFS (significance level: *p* ≤ 0.05).

## 3. Results

### 3.1. Study Population and Data

A total of 45 female outpatients treated with palbociclib for metastatic breast cancer were enrolled in this study. One patient (n = 7 concentrations) with known severe adherence issues, which would prevent us from reconstructing a precise history of palbociclib intake, was excluded from the analysis. Table 1 presents the characteristics of the study population. Overall, two concentrations were excluded from the analysis due to poor confidence in date of last drug intake (n = 1) and very low concentration (n = 1). The final population analysis included 255 palbociclib plasma concentrations (range: 2.0–159.0 ng/mL) collected at unselected timing after last dose intake (from 5 min to 255 h after last dose, with samples also collected during the OFF-treatment period) (electronic Supplementary Material, Figure S1). A median of 5 concentrations [range 1–16] was collected per patient. The median daily dose was 100 mg, varying from 75 to 125 mg.

A total of 1174 ANC values, 40 of which were collected before palbociclib initiation, were available for the exposure–toxicity modeling.

### 3.2. Pharmacokinetic Model and Exposure–Toxicity Relationship

#### 3.2.1. Pharmacokinetic Analysis

##### Base Model

Palbociclib plasma concentrations were best described by a two-compartment model with first-order absorption including a lag time. The addition of a second compartment significantly improved the fit (∆OFV = −17.3, *p* < 0.001). Inter-individual variabilities were significant on k_a_ and V_c_/F (∆OFV < −24.9, *p* < 0.001) in addition to CL/F. A proportional error model adequately captured the residual variability.

##### Covariate Analysis

No impact of PPI co-administration alone was found (∆OFV > −0.36, *p* > 0.05). However, palbociclib CL/F was increased by 56% when palbociclib was taken under fasting conditions (or with a light meal) and simultaneously administered with PPI (∆OFV = −12.3, *p* < 0.001). None of the other covariates significantly affected palbociclib PK parameters (∆OFV > −5.65, *p* > 0.005) after backward deletion. Parameter estimates of the final palbociclib PK-only model along with associated bootstrap results are presented in Table S1.

##### External Validation

A total of 71 additional palbociclib concentrations were included in the external validation: 35 concentrations from 26 patients enrolled in the OpTAT study complemented by 36 measurements from 19 patients of the Hospital Cochin. The model adequately predicted palbociclib concentrations in the validation dataset, with a non-significant bias of 2% (CI_95%_ −3% to 8%) and a precision of 32%.

#### 3.2.2. Exposure–Toxicity Relationship

##### Base Model

**Figure 1:**
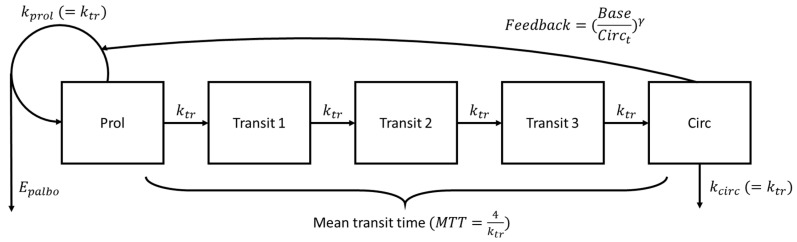
Semi-mechanistic PK/PD structural model describing neutropenia after palbociclib administration [18]. $E_{palbo}$: palbociclib effect parameter described by an *E_max_* model, $k_{prol}$ : net proliferation rate constant, $k_{tr}$ : transit rate constant, $k_{circ}$ : physiological neutrophil elimination rate constant from systemic circulation, MTT: mean transit time through the maturation delay chain, Prol: proliferating progenitor cells, Circ: circulating cells, Base : baseline value of circulating cells, $Circ_{t}$ : circulating cells at a given time t, γ: power factor for the feedback mechanism.

The semi-mechanistic neutropenia model shown in Figure 1 successfully described the ANC time course. The typical base ANC value, i.e., prior to palbociclib initiation, was 4.1 G/L, representative of a normal neutrophil count (2 to 7.5 G/L). The E_max_ model was superior to the linear one (∆AIC = −76), but the estimated EC_50_ value did not match the literature value (8.8 vs. 40.1 ng/mL [19]). This difference was attributed to data sparseness in our study along with the narrow dose interval preventing us from adequately estimating this parameter. Therefore, EC_50_ was fixed at the literature value (40.1 ng/mL [19]). Inter-individual variabilities were estimated on MTT, E_max_, and EC_50_ (∆OFV < −58.5, *p* < 0.001) in addition to the base ANC.

##### Covariate Analysis

Univariate analyses revealed that baseline GGT had a significant effect on the base ANC value, and eGFR significantly affected E_max_ (∆OFV < −6.3, *p* < 0.05). After backward deletion, none of the covariates was retained in the final model. Table 2 presents the final model parameters when estimating both PK and PD parameters. All parameters were estimated with good precision (RSE ≤ 47%).

##### Validation of the Final Exposure–Toxicity Model

Individual ANC fits demonstrated that the final model adequately described ANC’s time course under palbociclib treatment (data not shown). Goodness-of-fit plots, shown in Figure S2, did not evidence any significant prediction bias.

**Figure 2:**
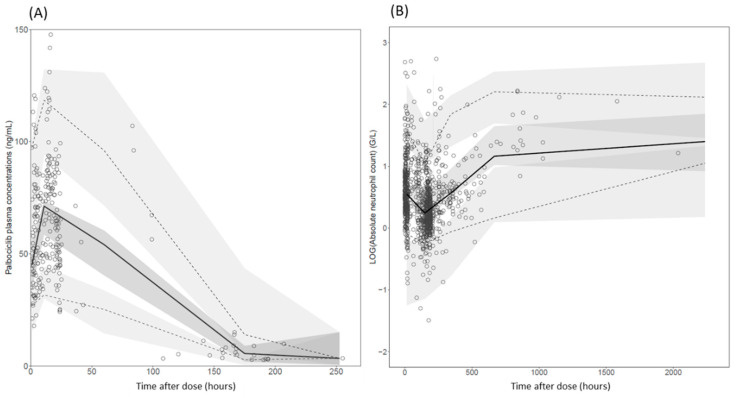
pc-VPC for the final PK-only (**A**) and the final PK/PD model (**B**). Open circles represent palbociclib plasma concentrations (**A**) and log transformed ANC (**B**). The continuous line shows the median observed values, and the dashed lines represent the observed 5% and 95% percentiles. Shaded areas show the model-based 95% confidence interval for the median and the percentiles.

Bootstrap results are presented in Table 2. The model was judged accurate and robust since parameter estimates were contained within the bootstrap 95% confidence interval (95% CI). The pc-VPCs showed that the simulated data adequately matched the observed values for both PK and PD data (Figure 2).

#### 3.2.3. Model-Based Simulations

##### PPI Effect

**Figure 3:**
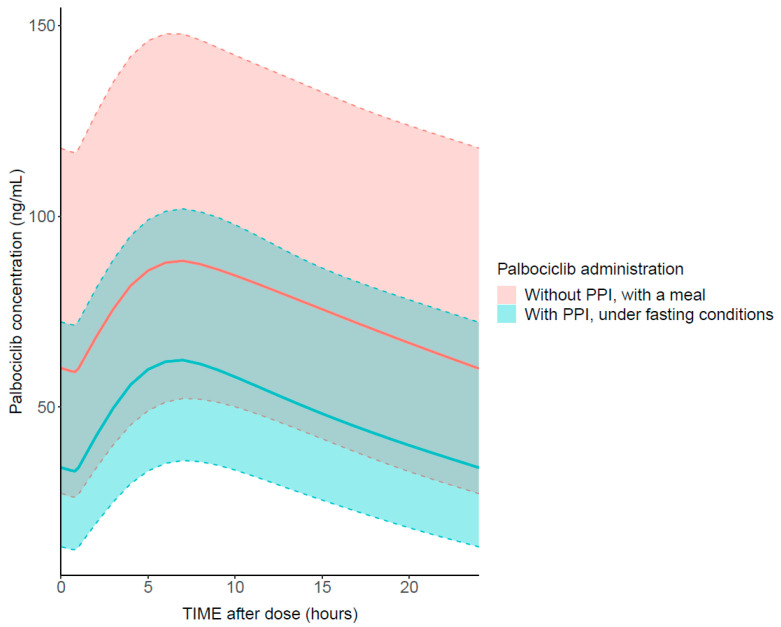
Steady-state palbociclib simulated plasma concentrations in 1000 individuals receiving a standard dose of 125 mg once daily under two different conditions (palbociclib intake with a meal and without PPI versus under fasting conditions with PPI). Continuous lines show the population median prediction, and shaded areas represent the 95% prediction intervals.

Simulations based on the final PK model (i.e., including the covariate effect on palbociclib CL/F) showed a 35% lower median exposure in patients taking palbociclib under fasting conditions with a PPI compared to those taking palbociclib during a meal without PPI (Figure 3).

##### Neutropenia Characteristics

**Figure 4:**
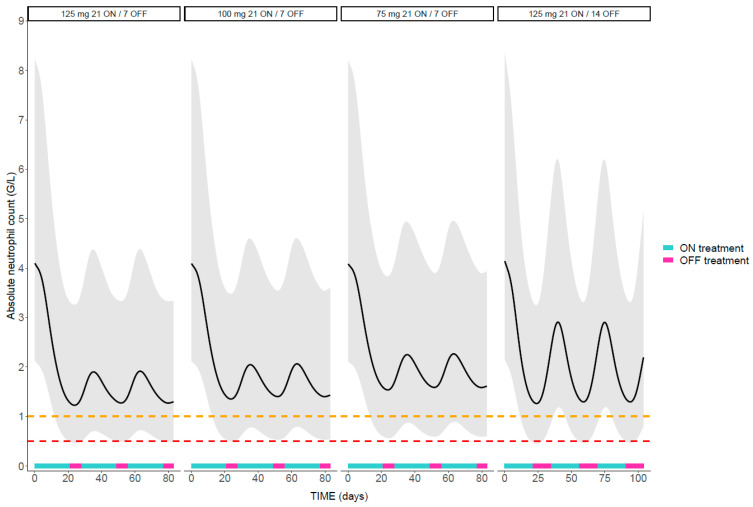
Model-based simulations of ANC dynamics following 3 cycles of different palbociclib dosage regimens (125, 100, or 75 mg, and 7- or 14-days OFF-treatment periods). The orange and red dotted lines represent the ANC threshold for grade 3 and 4 neutropenia, respectively. The black lines represent the population median prediction, and the shaded areas show the 95% prediction intervals.

Model-based simulations indicated that after the initiation of a standard palbociclib administration schedule, ANC decreases and reaches a nadir after 24 days (i.e., 21 days ON followed by 3 days OFF), regardless of the dose (i.e., 125, 100, or 75 mg) [interquartile range IQR 24–25] (Figure 4). Palbociclib-induced neutropenia is reversible, with a rebound occurring during the following 11 days (i.e., last 4 days OFF/first 7 days ON treatment of the subsequent cycle). Palbociclib-induced neutropenia appeared non-cumulative since the same pattern was then observed during each subsequent cycle, with no trend of worsening ANC values over repeated cycles, in contrast to chemotherapy-induced neutropenia [26]. A higher incidence of neutropenia and lower ANC nadir is expected in patients with a low base ANC. Indeed, neutropenia grade 4 occurred in 0.4% and 16% of the simulated individuals with the highest and lowest base ANC values, respectively.

##### Influence of Palbociclib Dosage Regimen on Neutropenia

A higher incidence of neutropenia grade 3–4 was observed in patients receiving higher doses of palbociclib with the same administration schedule (i.e., 21 days ON/7 days OFF). Overall, 36%, 29%, and 21% of simulated patients receiving respectively 125, 100, or 75 mg of palbociclib experienced grade 3 neutropenia. These proportions decreased to 5%, 3%, and 2% for grade 4 neutropenia at the same doses.

The ANC nadir was not affected by dose, with similar values between palbociclib doses (0.8 G/L for grade 3 and 0.4 G/L for grade 4 neutropenia). The simulations of 125 mg 21 days ON/7 days OFF when palbociclib was taken under fasting conditions with a PPI provided similar results in terms of incidence and nadir compared to those for 75 mg 21 days ON/7 days OFF when palbociclib was taken during a meal without PPI (data not shown). An alternative administration schedule with a longer OFF-treatment period (i.e., 21 days ON/14 days OFF) while maintaining the same dose (i.e., 125 mg) demonstrated that a better ANC recovery was obtained, even though the base ANC value was not yet reached 14 days after the last dose. Following three palbociclib cycles, a median of 25 days [IQR 22–28 days] after the last palbociclib dose was necessary for ANC to recover to the base value for all simulated patients.

##### Exposure Threshold to Limit the Risk of Severe Neutropenia

**Figure 5:**
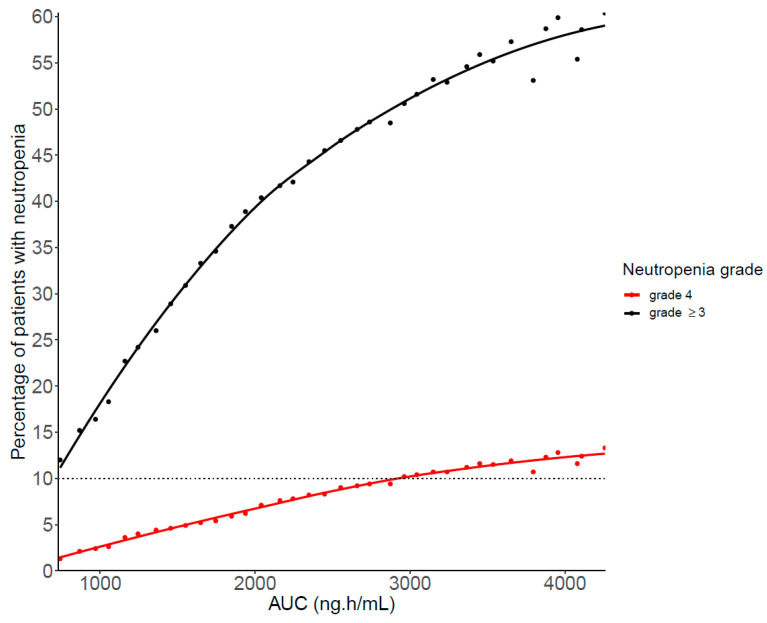
Incidence of grade ≥ 3 or grade 4 neutropenia according to palbociclib AUC_0–24_. The dotted line represents the empirical value of 10% of patients at risk of severe neutropenia as a maximum threshold not to be exceeded.

The risk of experiencing high-grade neutropenia increased with AUC_0-24_, as exemplified in Figure 5. Targeting an AUC_0–24_ lower than 2900 ng·h/mL would limit the risk of developing grade 4 neutropenia to 10%.

### 3.3. Exposure–Response Relationship

**Figure 6:**
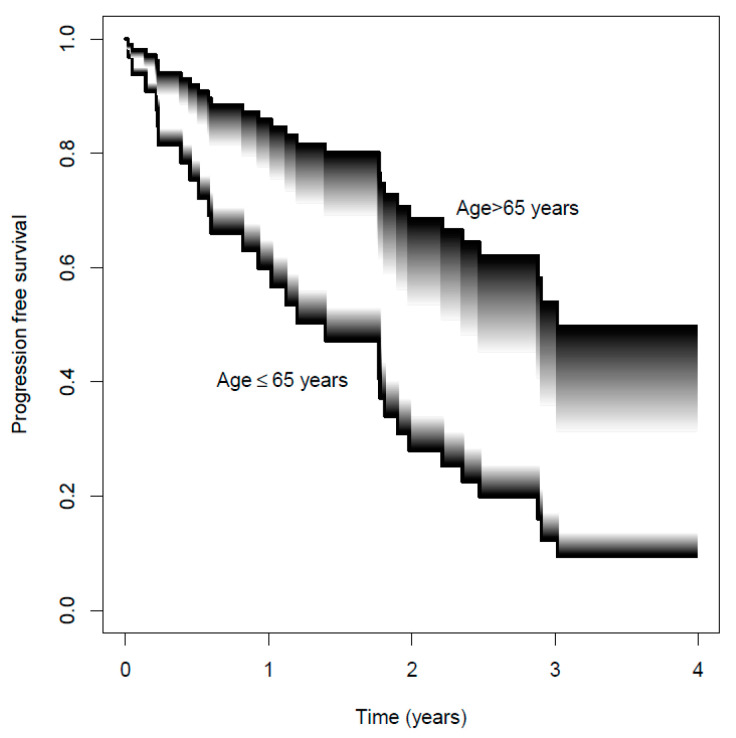
Survival functions predicted by the Cox model. From gray to black, AUC_cum90_ increases from the lower bound of the IQR (880 ng·h·mL^−1^) to its upper bound (1412 ng·h·mL^−1^). The bottom and top curves represent the survival functions in the younger (age ≤ 65 years, n = 25) and older (age > 65 years, n = 19) patients, respectively.

Among the 44 patients included, 29 progressed with a median PFS of 17 months (IQR 8–27). Overall, the median AUC_cum90_ was 1145 ng·h·mL^−1^ (IQR 880–1412 ng·h·mL^−1^). Results from the multivariable Cox analysis are represented in Table S2, and Figure 6 shows the survival functions on varying the AUC_cum90_ in the IQR and stratified by age (cutoff 65 years). Older age was the only covariate significantly associated with larger PFS (hazard ratio 0.42, *p* = 0.048). A trend to better PFS with increasing palbociclib exposure in older patients was observed (hazard ratio 0.88, *p* = 0.27). 

## 4. Discussion

Our study describes palbociclib PK/PD relationships in a real-world setting of patients with advanced breast cancer. The availability of some plasma concentrations measured during OFF-treatment days allowed the addition of a second compartment in the model and, consequently, a better description of palbociclib elimination. PK parameters are in good accordance with the model developed by the manufacturer [27] but differ from those estimated in real-world conditions [7,13]. The availability of rich concentration–time profiles in our study allowed us to confidently estimate all the PK parameters. Our study also highlights the high variability in palbociclib PK, consistently with results reported by Royer et al. [7]. Regarding palbociclib intake, our results support the manufacturer’s data not contraindicating concomitant administration of PPIs when palbociclib is taken with a meal. Indeed, the co-administration of PPIs when palbociclib is taken in fasting conditions decreased palbociclib exposure by 35%, a significant, although lower, effect compared to the 62% decrease in AUC described in the literature in a much less heterogeneous population of healthy volunteers [8]. However, the palbociclib intake with food and a simultaneous administration of PPIs did not result in any significant effect. None of the other covariates tested on the PK model was significant, while previously published studies showed a significant impact of renal (i.e., eGFR) [7,13] and hepatic (i.e., ALK) [13] functions on palbociclib PK. This might arise from the absence of extremes values (Table 1) compared to other studies, compromising our power to detect these effects.

Regarding our exposure–toxicity model, the estimated PD parameters are aligned with the literature values [13,19]. Due to data sparseness, EC_50_ was fixed to the value estimated in a previously published model, in which palbociclib doses from 25 to 225 mg allowed a reliable estimate of the drug effect parameters [19]. Model-based simulations confirmed that the incidence of neutropenia is dose and concentration dependent, supporting the dose reduction clinically implemented in patients experiencing neutropenia in a real-world setting. In addition, palbociclib-induced neutropenia is rapidly reversible, in contrast to that caused by more conventional chemotherapeutic agents. This is attributed to their different mechanism of action on bone marrow suppression, with palbociclib inducing a quiescence of progenitor cells without apoptosis, while chemotherapeutic agents cause DNA damage and apoptotic cell death [28]. The reversibility of neutropenia was exemplified by model-based simulations showing that ANC levels recovered after treatment withdrawal during the OFF-treatment periods. In the PALOMA-3 trial having enrolled patients receiving palbociclib (125 mg 21 days ON/7 days OFF) and fulvestrant, the median duration of grade ≥ 3 episodes was 7 days [29]. This is in accordance with our results (i.e., 8 days in the second cycle) although this time varied widely (IQR 3–15) between patients due to the large inter-individual variability of PD parameters. Interestingly, our model-based simulations highlighted the lag time between the start of the OFF-treatment period and the ANC rebound. Similarly, the resumption of treatment at each consecutive cycle does not coincide with the beginning of the ANC decrease. This is attributed to time of the maturation process of the precursor cells and of the feedback mechanism from the circulating neutrophils [18]. It is important that clinicians integrate this lag time into their clinical evaluation to avoid unnecessary anticipations of dose reductions or extensions of OFF-treatment periods when blood samples are collected while ANC has not yet started to recover. Finally, neutropenia under palbociclib treatment appears noncumulative, since no trend of worsening ANC over cycles was detected, as previously reported [19].

The large inter-individual variability in both PK and PD parameters argues for the optimization of management strategies and for the individualization of the regimen in terms of dosing and length of each cycle to decrease toxicity. Although febrile neutropenia is uncommon in patients receiving palbociclib [30], it is important to individually assess the risk of neutropenia to avoid treatment discontinuation. Given the model parameters, patients’ characteristics, and measured data (palbociclib concentration and ANC), a posteriori dosage adjustments and cycle delays can be proposed to minimize the risk of neutropenia in a real-world setting. Model-based simulations also provide guidance for the monitoring of palbociclib-induced neutropenia. Our data suggest that the current guidelines should be revisited, indicating that complete blood count monitoring should be performed at least prior to the start of each cycle and on day 14 of the first two cycles [31]. Simulations revealed that the ANC nadir occurred approximately at day 24 of each cycle. Therefore, when monitoring ANC on day 14, clinicians should be aware that ANC will continue to decrease during the following 10 days. According to our results, day 24 (3 days after the beginning of the OFF-treatment period) would be the optimal timing for hematologic monitoring to take rational clinical decisions regarding dose adjustments or cycle delays. This recommendation is valid when assuming an optimal medication adherence and highlights the importance of monitoring the actual cycle dates. In addition, it is important to monitor the ANC value at day 1 of each cycle to decide on the resumption of the treatment.

As opposed to other anticancer drugs such as imatinib with well-established therapeutic targets [32], no formal exposure target exists for palbociclib. Recently, Mueller-Schoell et al. suggested the use of the mean trough concentration (C_min_) observed in clinical trials (i.e., 61 ng/mL) as a target for exploratory therapeutic drug monitoring [33]. Comparing palbociclib concentrations measured in a patient to the expected values across the population might help to identify patients suspected of low adherence to treatment. It is indeed well known that treatment with intermittent dosing schedules could be more difficult to implement, which may affect adherence to medication and consequently drug PK and PD [34]. However, the proposed threshold does not reflect the risk of developing toxicity. Recently, Le Marouille et al. refined this target by suggesting an upper limit of 100 ng/mL for trough concentration to avoid severe neutropenia based on PK/PD modeling [13]. Regarding our exposure–toxicity model, we suggest that an AUC_0–24_ of 2900 ng·h/mL should not be exceeded to avoid a risk of grade 4 neutropenia higher than 10%. In our opinion, AUC_0–24_ better reflects the entire PK profile that influences the risk of neutropenia compared to C_min_. The same trough concentration can be measured for several patients with diverse PK profiles and, thus, with different risk of developing neutropenia. Conversely, a more homogeneous risk of neutropenia is expected across patients with the same AUC_0–24_. Although for clinicians AUC_0-24_ is more difficult to apprehend compared to C_min_, such a metric can be obtained for each patient using tools based on the Bayesian approach. This is proposed in the Tucuxi software developed by the University Hospital of Lausanne and the School of Management and Engineering Vaud [35], and in the non-parametric BestDose software (http://www.lapk.org/bestdose.php (accessed on 20 January 2022)). If our results are confirmed, therapeutic drug monitoring could guide clinicians to rapidly define the optimal individual dosage regimen. In practice, this would improve the quality of life of the patients by avoiding repeated medical visits and, thus, decreasing the medical burden.

Concerning the exposure–efficacy relationship, the positive effect of older age on PFS can be explained by the slower rate of cell development and higher frequency of less aggressive cancers in older compared to younger people, as already reported [36]. We did not evidence any significant association between AUC_cum90_ and progression. Nevertheless, a trend for better PFS with increasing palbociclib exposure was observed in older patients. Data from the manufacturer indicated a trend for better PFS with higher exposure, but the limited sample size and the fixed dose of 125 mg prevented a reliable estimation of the PK/PD relationship [4]. In other studies, no significant association between PFS and average palbociclib concentrations [37] or varying doses [11,38] was evidenced.

This study presents some limitations that should be acknowledged. The major limitation arises from shortcoming of the data, which prevented us from estimating the EC_50_ parameter and drawing definitive conclusions on the exposure–response relationship. In addition, some covariates’ effects were not tested due to the limited number of observations under specific conditions (i.e., only 1% of palbociclib concentrations were measured when co-administered with antacids such as magnesium or aluminum compounds; only 0.6% of ANC values were measured during concomitant radiation therapy). Nevertheless, the strengths of the study should be emphasized. To our knowledge, this is the first study evaluating exposure–toxicity along with exposure–response relationships of palbociclib using real-world data, providing valuable insights onto drug management in clinical practice.

In conclusion, the semi-mechanistic PK/PD model adequately described ANC time courses observed in a real-world population of patients receiving palbociclib for the treatment of advanced breast cancer. We showed that an increase in palbociclib exposure resulted in a higher incidence of neutropenia and proposed an exposure threshold not to be exceeded to avoid severe neutropenia. However, we did not evidence any significant association between exposure and PFS. These results indicate that the palbociclib dose might be reduced in patients experiencing severe neutropenia without significantly compromising efficacy. A thorough understanding of the exposure–toxicity and exposure–response relationships may guide clinicians to optimize their management strategies and to individually adjust dosing regimens.

## Figures and Tables

**Table 1:** Demographic and clinical characteristics of the study population.

Patients’ Characteristics	Median [IQR] or n (%)	Missing Data (%)
Age (years) ^1^	65 [55–75]	
Body weight (kg) ^1^	67 [61–80]	
AST (UI·L^−1^) ^1^	23 [19–28]	8
ALT (UI·L^−1^) ^1^	20 [15–27]	9
BILT (µmol·L^−1^) ^1^	5 [4–7]	13
Albumin (g·L^−1^) ^1^	43 [41–45]	26
ALK (UI·L^−1^) ^1^	61 [49–81]	9
GGT (UI·L^−1^) ^1^	29 [18–51]	20
CRT (µmol·L^−1^) ^1^	71 [63–85]	9
eGFR (mL·min^−1^·1.73 m^−2^) ^1^	71 [54–98]	9
Number of cycles per patient	33 [22–38]	
Administration under fasting conditions ^2^	54 (21)	
Co-administration of weak and moderate CYP3A4 inhibitor ^2^	44 (17)	
Co-administration of weak and moderate CYP3A4 inducer ^2^	13 (5)	
Co-administration of PPI ^2^	78 (31)	
Co-administration with fulvestrant ^3^	822 (70)	
Chemotherapy in the previous treatment line ^3,^*	256 (22)	
POCT measurement ^3^	295 (25)	

Values are reported at the time of ANC and palbociclib measurements ^1^, according to the number of palbociclib concentrations ^2^, and according to the number of ANC measurements ^3^. * Chemotherapeutic agents received in the previous treatment line included capecitabine (n = 1), capecitabine and ixabepilone (n = 1), carboplatin and doxorubicin (n = 1), carboplatin and paclitaxel (n = 1), cisplatin and gemcitabine (n = 1), doxorubicin (n = 1), eribulin (n = 2), paclitaxel (n = 1), and paclitaxel co-administered with bevacizumab (n = 3). ALK: alkaline phosphatase, ALT: alanine aminotransferase, AST: aspartate amino transferase, BILT: total bilirubin, CRT: creatinine, eGFR: estimated glomerular filtration rate, GGT: gamma-glutamyltransferase, POCT: point of care test, PPI: proton-pump inhibitor.

**Table 2:** Parameter estimates in the final PK/PD model with bootstrap results.

Parameter	Final Model		Bootstrap (n = 502 Samples)	
	Estimate	RSE (%)	Median	CI_95%_
Pharmacokinetics				
k_a_ (h^−1^)	0.8	30	1.0	0.5–3.0
ω_ka_ (CV%)	125	26	120	5–368
ALAG (h)	2.0	8	2.0	1.8–2.5
CL/F (L·h^−1^)	67	5	68	62–74
ω_CL_ (CV%)	29	12	28	20–35
CL/F_PPI,no food_ (L·h^−1^)	131	4	132	114–151
V_c_ /F(L)	2800	7	2778	2295–3256
ω_Vc_ (CV%)	32	16	28	5–41
Q (L·h^−1^)	7	31	7	4–25
V_p_/F (L)	704	9	738	629–1376
Proportional residual error (%)	18	9	18	15–21
Pharmacodynamics				
Base (G·L^−1^)	4.1	7	4.1	3.6–4.7
ω_base_ (CV%)	35	14	35	25–44
MTT (h)	122	5	121	109–133
ω_MTT_ (CV%)	12	28	12	2–19
E_max_	0.22	7	0.22	0.19–0.25
ω_Emax_ (CV%)	15	31	16	1–25
EC_50_ (ng·mL^−1^)	40.1	FIX		
ω_EC50_ (CV%)	93	47	91	1–529
γ	0.13	9	0.13	0.11–0.17
Additive residual error ^a^	0.31	9	0.30	0.26–0.35

ALAG: absorption lag time, base: baseline level of circulating absolute neutrophil counts, CI: confidence interval, CL/F: apparent clearance, CL/F_PPI,no food_: apparent clearance under fasting conditions with concomitant co-administration of PPI, CV: coefficient of variation, EC_50_: palbociclib concentration corresponding to 50% of the maximum effect, E_max_: maximum estimated drug effect, k_a_: absorption rate constant, MTT: mean transit time, Q: inter-compartmental clearance, RSE: relative standard error, V_c_/F: apparent central volume of distribution, V_p_/F: apparent peripheral volume of distribution, ω: between-subject variability, γ: feedback parameter. ^a^ additive residual error in log scale.

## Data Availability

The data presented in this study are available on request from the corresponding author. The data are not publicly available due to ethical reasons.

## Supplementary Materials

The following supporting information can be downloaded at https://www.mdpi.com/article/10.3390/pharmaceutics14071317/s1: Figure S1: Normalized observed palbociclib plasma concentrations-time profiles. Concentrations are normalized for a daily dose of 125 mg. Concentrations from patients with rich sampling are joined with black lines, Figure S2: Goodness-of-fit plots of the final model for palbociclib concentrations (A) and ANC time course (B). Loess smooth curves are printed in grey. (a) Observed concentrations vs. individual predictions; line of identity is printed in black. (b) Observed concentrations vs. population predictions; identity line is printed in black. (c) Conditional weighted residuals (CWRES) vs. population predictions; ordinate value zero line is printed in black. (d) CWRES vs. time post-dose; ordinate value zero line is printed in black, Table S1: Parameter estimates of the final palbociclib PK-only model with bootstrap results, Table S2: Results from the Cox analysis.
