# Supplementary material for: Population Pharmacokinetics of Palbociclib and Its Correlation with Clinical Efficacy and Safety in Patients with Advanced Breast Cancer
Source: Pharmaceutics. 2022 Jun 21;14(7):1317. doi: 10.3390/pharmaceutics14071317 (PMC9322950; doi:10.3390/pharmaceutics14071317)
Supplement: Supplementary file 1 [file pharmaceutics-14-01317-s001.zip › pharmaceutics-1738985-SM.pdf]

Electronic supplementary material

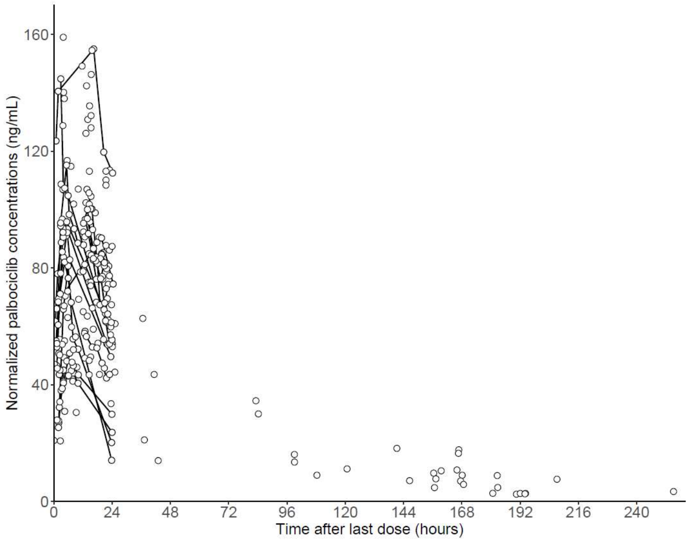

Figure S1. Normalized observed palbociclib plasma concentrations-time profiles. Concentrations are normalized for a daily dose of 125 mg. Concentrations from patients with rich sampling are joined with black lines.

(A)

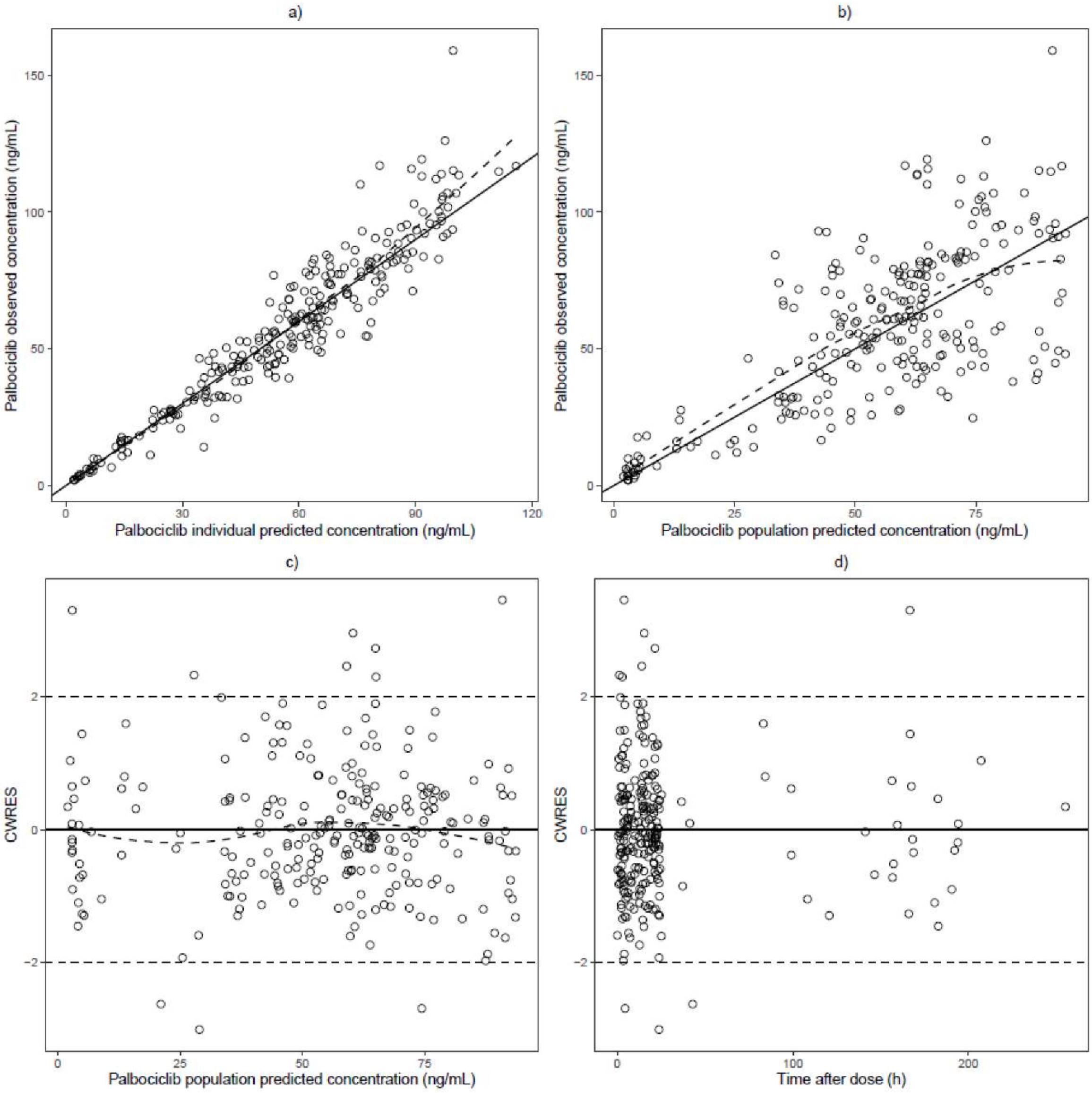

a)
b)
c)
d)
Palbociclib observed concentration (ng/mL)
Palbociclib individual predicted concentration (ng/mL)
Palbociclib population predicted concentration (ng/mL)
CWRES
Time after dose (h)
0
30
60
90
120
50
100
150
25
75
200
2
-2

(B)

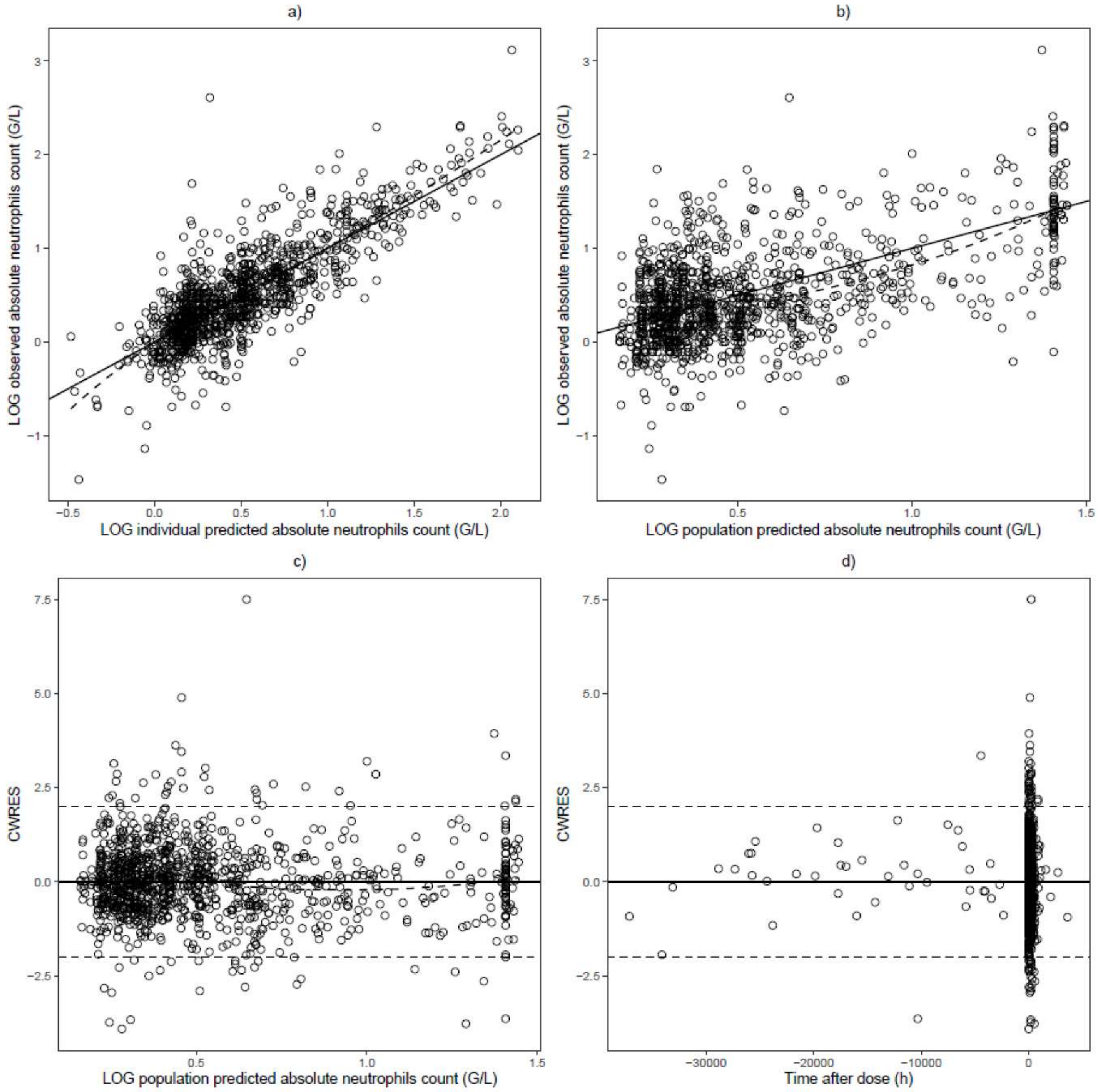

Figure S2: Goodness-of-fit plots of the final model for palbociclib concentrations (A) and ANC time course (B). Loess smooth curves are printed in grey. a) Observed concentrations vs. individual predictions; line of identity is printed in black. b) Observed concentrations vs. population predictions; identity line is printed in black. c) Conditional weighted residuals (CWRES) vs. population predictions; ordinate value zero line is printed in black. d) CWRES vs. time post-dose; ordinate value zero line is printed in black

Table S1: Parameter estimates of the final palbociclib PK-only model with bootstrap results.

Parameter	Final model		Bootstrap (n=2000 samples)	
	Estimate	RSE (%)	Median	$CI_{95\%}$
k_a (h^{-1})	0.73	34	0.78	0.39-3.27
ω_{ka} (CV%)	126	25	121	33-458
ALAG (h)	1.9	4	1.9	1.7-2.6
CL ($L.h^{-1}$)	68	5	68.0	61-75
ω_{CL} (CV%)	30	12	29	22-36
$CL_{IPP,no\ food}$ ($L.h^{-1}$)	106	9	106	88-144
V_c (L)	2730	10	2700	2136-3229
ω_{Vc} (CV%)	36	24	34	14-56
Q ($L.h^{-1}$)	5.1	43	5.2	3.2-23.7
V_p (L)	717	10	807	655-2931
Proportional residual error (%)	18	19	17	14-20

ALAG: absorption lag-time, CI: confidence interval, CL: clearance, $Cl_{IPP,nofood}$: clearance with concomitant coadministration of IPP under fasting conditions, CV: coefficient of variation, k_a: absorption rate constant, RSE: relative standard error, Q: inter-compartmental clearance, V_c: central volume of distribution, V_p: peripheral volume of distribution, ω: between subject variability.

Table S2: Results from the Cox analysis.

Parameter	Coefficient	Hazard ratio	*p*-value
AUC_{cum90}	0.030	1.030	0.682
Older age (>65 years)	-0.872	0.418	0.048
Interaction AUC_{cum90}*older age	-0.125	0.883	0.266

Results are presented for an increase in 100 AUC units.
